# Case Report: Unresectable pulmonary metastases of a giant cell tumor of bone treated with denosumab: a case report and review of literature

**DOI:** 10.3389/fonc.2023.1230074

**Published:** 2023-08-16

**Authors:** Shinji Miwa, Norio Yamamoto, Katsuhiro Hayashi, Akihiko Takeuchi, Kentaro Igarashi, Yuta Taniguchi, Sei Morinaga, Yohei Asano, Takayuki Nojima, Hiroyuki Tsuchiya

**Affiliations:** ^1^ Department of Orthopedic Surgery, Graduate School of Medical Sciences, Kanazawa University, Kanazawa, Japan; ^2^ Department of Pathology, Graduate School of Medical Sciences, Kanazawa, Japan

**Keywords:** metastasis, giant cell tumor of bone, PET, denosumab, unresectable

## Abstract

Giant cell tumors of bone (GCTB) sometimes metastasize to distant organs. In this case report, we present pulmonary metastases of GCTB mimicking malignancies. A 49-year-old man underwent two surgical treatments for a GCTB of the right proximal radius. At the time of the second surgery, no lesions were observed on chest radiography. Three years after surgery, the patient presented with cough and dyspnea, and chest radiography and computed tomography (CT) revealed multiple lung nodules. Positron emission tomography/CT revealed a high accumulation of 18F-fluoro-2-deoxy-D-glucose (18F-FDG) in multiple lesions. Based on the rapid growth and accumulation of 18F-FDG, a metastatic malignant tumor was suspected. CT-guided needle biopsy was performed, and the histology showed proliferation of spindle cells and multinuclear giant cells without malignant changes. Denosumab was administered because multiple lung lesions were unresectable. One month after denosumab treatment, CT showed marked shrinkage of the lesions, and the symptoms significantly improved. Eighteen months after the initial treatment with denosumab, the patient had no symptoms or tumor growth. Although its long-term efficacy and safety remain unclear, denosumab may be a treatment option for patients with unresectable pulmonary GCTB.

## 1 Introduction

Giant cell tumor of bone (GCTB), a locally aggressive and rarely metastasizing tumor, is classified as intermediate malignancies according to the 2020 World Health Organization (WHO) classification (1–3). Histologically, GCTB consist of ovoid mononuclear cells and giant osteoclast-like cells (4, 5). Osteoclast-like multinuclear cells and their precursors express receptor activator of nuclear factor-kappa B (RANK), whereas mononuclear stromal cells express RANKL, which is necessary for the formation, function, and survival of the osteoclasts (6–9).

The standard treatment for GCTB is surgical excision, which consists of curettage and en bloc resection (10). In cases of unresectable GCTB, denosumab, a human monoclonal antibody targeting the nuclear factor-kappa B ligand (RANKL), is considered a treatment option (5, 11, 12). Denosumab binds to RANKL and blocks its binding to RANK on osteoclasts and osteoclast precursors, resulting in the inhibition of osteoclast differentiation and bone resorption by the osteoclasts (5, 12). Denosumab is widely used to prevent hypercalcemia, pathological fractures, and spinal cord compression in patients with metastatic bone diseases (13–15). Furthermore, high response rates have been reported in clinical studies on denosumab in patients with GCTB (5, 11). In a phase 2 study of denosumab in patients with GCTB, patients received 120 mg of subcutaneous denosumab (every 4 weeks with a loading dose on days 8 and 15 of the first cycle) (16). In this study, 163 of 169 (96%) patients were progression-free after a median follow-up of 13 months (16). In another study including 43 patients with resectable GCTB and 54 patients with unresectable GCTB, all tumors were controlled by denosumab treatment, whereas 40% of patients who discontinued denosumab showed tumor progression after a median of 8 months (12). Furthermore, it is reported that neoadjuvant treatment with denosumab can downstage the lesions by increasing the thickness of cortical bone and forming new cortical rim around the soft-tissue mass that facilitates joint salvage and decrease surgery invasiveness (1, 17–23). Although denosumab does not have direct cytotoxic effect on neoplastic stromal cells, it can inhibit pulmonary metastases. Denosumab prevents RANKL-mediated formation and activation of multinucleated giant cells from RANK-positive mononuclear preosteoclasts and macrophages, resulting in marked reduction in multinucleated giant cells (5, 24–26). Although denosumab is considered an effective treatment option for patients with unresectable GCTB, the indications, doses, and periods of denosumab use in metastatic GCTB remain unclear. Therefore, investigations into treatment strategies for unresectable metastatic GCTB are required.

In the management of malignant tumors, ^18^F-fluoro-2-deoxy-D-glucose (^18^F-FDG) positron emission tomography/computed tomography (PET/CT) is one of the most useful diagnostic tools to assess grading, staging, therapeutic response, surgical planning, and expected prognosis (27). PET/CT can be used to differentiate between malignant and benign lesions (28). However, GCTB has high accumulation of ^18^F-FDG (27), and the high accumulation of ^18^F-FDG may mislead the diagnosis of the tumor (29–31). In this report, we present a case of pulmonary metastasis of GCTB that mimicked malignancies on radiographic examinations, which was successfully controlled by treatment with denosumab.

## 2 Case presentation

**Figure 1:**
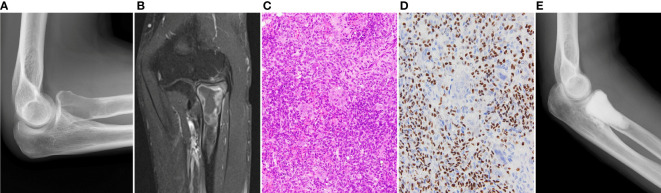
**(A)** Initial radiography shows an osteolytic lesion in the proximal radius. **(B)** In enhanced magnetic resonance imaging, the lesion is enhanced by gadolinium. **(C)** Histology of the specimen of the radius shows proliferation of spindle cells and multinucleated osteoclast-like cells, which is consistent with diagnosis of GCTB. **(D)** Immunohistological staining of H3.3G34W. **(E)** The lesion is curetted and augmented with α-tricalcium phosphate.

**Figure 2:**
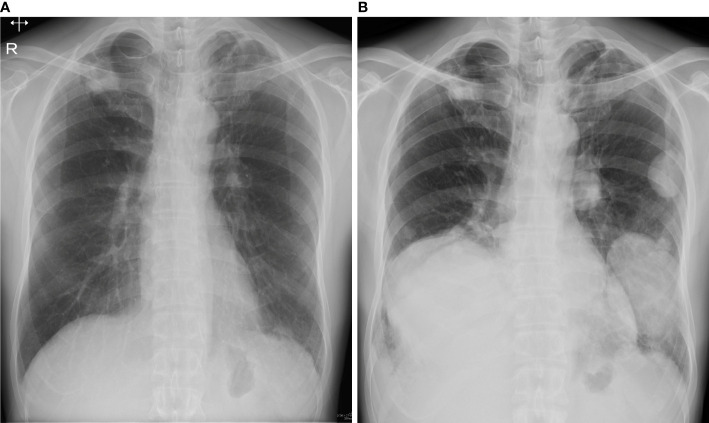
**(A)** At the time of second surgery, no pulmonary lesion was detected on radiography. **(B)** Three years after the second surgery, radiography showed multiple lung nodules.

A 49-year-old man presented with right elbow pain. Radiography revealed osteolytic lesions and scalloping in the right proximal radius (**Figure 1A**). Magnetic resonance imaging showed a tumor lesion in the proximal radius with iso intensity on T1-weighted images and high intensity on T2-weighted images, and the lesion was enhanced by gadolinium (**Figure 1B**). ^201^Thallium (^201^Tl) scintigraphy and ^99m^Tc-methylene diphosphonate (^99m^Tc-MDP) scintigraphy showed an increased uptake of the ^201^Tl and ^99m^Tc-MDP in the proximal radius. Open biopsy was performed, and the histology of the tumor showed proliferation of spindle cells and multinuclear osteoclast-like cells with collagen fibers and deposition of hemosiderin (**Figure 1C**). Immunohistological staining showed positivity for H3.3G34W in the tumor cells (**Figure 1D**). Based on the histological findings, the tumor was diagnosed as a GCTB. He underwent curettage with adjuvant treatment with ethanol and phenol, and the bone defect was augmented with α-tricalcium phosphate (αTCP) (**Figure 1E**). Two years after the initial surgery, radiography and CT revealed tumor recurrence. He underwent denosumab treatment (120 mg on days 1, 8, 15, 29, 56, and 84), curettage with adjuvant treatment with ethanol and phenol, and artificial bone grafting using α-TCP. Histological examination of the tumor specimen showed proliferation of spindle cells and multinuclear giant cells, consistent with GCTB recurrence. At the time of the second surgery, no pulmonary nodules were detected on chest radiography (**Figure 2A**).

**Figure 3:**
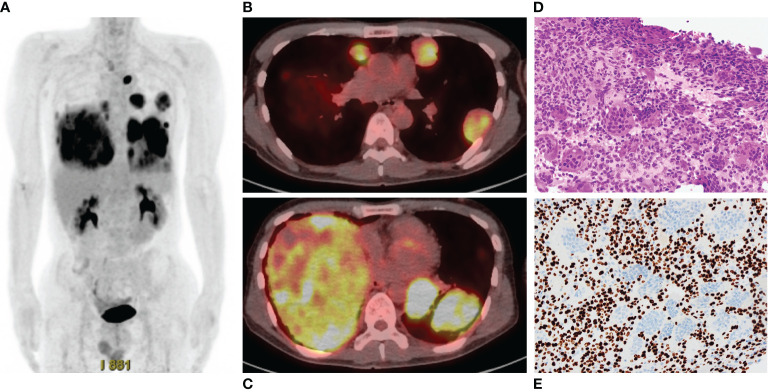
**(A)** Positron emission tomography/computed tomography shows high accumulation of ^18^F-fluoro-2-deoxy-D-glucose in the multiple lung lesions. **(B, C)**. The maximum standardized uptake values are 11.8–12.2. **(D)** Histology of specimen of the lung nodule shows proliferation of spindle cells and multinuclear cells, which is similar to the findings of specimen of the radius. **(E)** Immunohistological staining of H3.3G34W.

**Figure 4:**
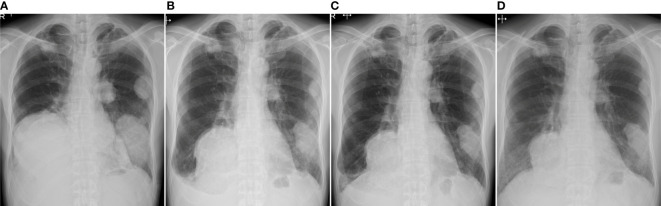
Radiography examination before treatment **(A)**, 1 month **(B)**, 3 months **(C)**, and 18 months **(D)** after the initial treatment of denosumab. The pulmonary lesions show response to the treatment of denosumab.

Three years after the second surgery, the patient presented with cough and dyspnea. Radiography and CT revealed 31 nodules, including the largest lesion (15 × 13 cm) in the lungs (**Figure 2B**). PET/CT revealed a markedly increased uptake of ^18^F-FDG in the lesions (SUVmax = 11.8–12.2) (**Figures 3A–C**). Based on the clinical course and radiological findings, malignant metastasis was suspected. CT-guided needle biopsy was performed to confirm the diagnosis of multiple lung lesions. Histological examination of the specimen revealed proliferation of spindle cells and multinuclear osteoclast-like cells without malignant changes (**Figure 3D**), and the tumor cells were positive for H3.3G34W on immunohistological staining (**Figure 3E**). These findings were similar to the histology of the primary lesions of the proximal radius, and the lung lesions were diagnosed as multiple metastases from GCTB. Because the lung nodules were thought to be unresectable, he was treated with denosumab (120 mg subcutaneously on days 1, 8, 15, and 29 and every 4 weeks thereafter). One month after the initiation of denosumab treatment, shrinkages of the lung nodules and gradual improvement in pulmonary symptoms were observed (**Figure 4**). Then, the denosumab treatment continued every 4 weeks. During the denosumab treatment, the patient underwent chest X-ray every month, blood examination every 3 months, and chest CT every 6 months. After 3 months of denosumab treatment, the patient achieved a partial response according to the Response Evaluation Criteria in Solid Tumors criteria (32). During the treatment 7period, X-ray and CT showed no regrowth of the tumor, and there were no adverse events, such as hypocalcemia and osteoporosis of the jaw. Eighteen months after the initial treatment with denosumab, the patient showed no symptoms or disease progression and continued to undergo denosumab treatment (**Figure 4**).

## 3 Discussion

In this report, we presented a case of multiple pulmonary metastases of GCTB. In this case, multiple pulmonary lesions were unresectable; however, these lesions were successfully controlled with denosumab treatment. The incidence of metastasis in GCTB has been reported to be 1–9% (33–37), and the lung is the most common site of metastasis, followed by the brain, kidney, bone, skin, and lymph nodes (34). Although most metastatic lung lesions of GCTB are slow-growing and can be controlled by tumor resection or observation (10, 33), some metastatic GCTB are aggressive and causes mortality (10, 38, 39). Although our follow-up protocol for patients with GCTB did not include chest X-ray, addition of chest X-ray or CT to the follow-up protocol is recommend to diagnose pulmonary metastases of GCTB earlier. Tsukamoto et al. reported that follow-up protocol after surgical treatment for GCTB included chest X-ray or CT every 4 months for the first 2 years, every 6 months for the next 3 years, and then annually (10). Chan et al. investigated the risk factors for pulmonary metastasis from GCTB in 291 patients with benign GCTB (39). In this study, only local recurrence was an independent risk factor for pulmonary metastases. Tsukamoto et al. investigated the outcomes and safety of initial observations in patients with pulmonary metastases of GCTB (10). In this study, 46% of patients with lung lesions ≤ 5 mm and all patients with lung lesions > 5 mm had disease progression. The patients with lung lesions ≤ 5 mm had significantly better progression-free survival than those with lung lesions > 5 mm (p = 0.022). Initial observation may be an option for patients with small pulmonary lesions. However, the present case required treatment because the pulmonary metastases were large and symptomatic.

In this case, clinical course and high accumulation of ^18^F-FDG in PET/CT mimicked malignancies. In previous reports, GCTB had higher accumulation of ^18^F-FDG (mean SUVmax = 8.4–16.8) than other benign lesions (28, 40–43). Uptake of FDG in tumor cells is associated with expression of glucose transporter protein (GLUT)-1, hexokinase II, and with gene upregulation for these proteins (44, 45). Ong et al. reported significantly greater GLUT-1 and hexokinase II in human cancer cell lines (46). On the other hand, other studies showed upregulation of GLUT-1 in monocyte-derived macrophages (47–49), and the high ^18^F-FDG uptake may be explained by high monocyte/macrophage content within GCTs (44, 50). Based on the reports, GCT and other lesions containing active macrophages should be considered in differential diagnoses of lesions with high accumulation of ^18^F-FDG.

There are several reports on systemic treatment for metastatic pulmonary GCTB (**Table 1**) (51–60). In a retrospective study of denosumab treatment in 7 patients with pulmonary GCTB, 3 patients showed partial response and 4 patients had stable disease (54). Based on the previous reports, denosumab can be one of the treatment options in patients with unresectable pulmonary GCTB (54, 56, 59, 60). However, denosumab can cause severe side effects, including hypocalcemia, hypophosphatemia, increased risk of atypical femoral fracture, and osteonecrosis of the jaw (5, 16). Therefore, long-term treatment with denosumab is not ideal therapeutic option, and discontinuation, dose reduction, or extension of the treatment interval, may be needed to avoid severe adverse effects of denosumab. Although denosumab can reduce tumor size by inhibiting osteoclastic differentiation and reducing giant cells, it is not cytocidal in the neoplastic stromal cells of GCTB, and discontinuation of the treatment may cause regrowth of the tumor (12). Tanikawa et al. reported a case treated with extended interval of the denosumab treatment for GCTB in the sphenoid bone (61). In their report, denosumab treatment (120 mg) for the first 2 years was performed every 4 weeks. Subsequently, the treatment interval was gradually extended, with 4 monthly dosing for the next 1 year, followed by a 6 monthly dosing for 2 years. During the extension of the treatment interval, slight growth of the lesion was observed, but it was thought to be acceptable range. They concluded that optimal extended dosing interval of denosumab treatment after achieving the stabilization of GCTB was 6 months. Thus, reducing the dose or extending the dosing interval as much as possible is desirable for patients who are unable to discontinue the medication.

**Table 1:** Recent reports of systemic treatment of pulmonary metastasis of GCTB.

Author	N	Treatment	Malignant change	Response	Severe side effect
Gong T (29)	1	Denosumab	–	PD	–
		Denosumab + apatinib		PR	
Feng L (30)	1	Denosumab and radiotherapy	–	Denosumab: PD Radiotherapy: PR	–
Wang G (31)	1	Denosumab	–	PD	–
		Denosumab + sunitinib		PR	
Luo Y (32)	7	Denosumab	–	PR: 3 SD: 4	–
Sachan DK (33)	1	Chemotherapy and radiotherapy	–	CR	–
Yamagishi T (34)	1	Denosumab	–	PR	–
Wei F (35)	2	IFN-α	–	PR	leukocytopenia
		IFN-α	–	PR	leukocytopenia
Iwai T (36)	1	Pazopanib	+	PR	–
Kudawara I (37)	1	Denosumab	–	PR	–
Egbert RC (38)	1	Denosumab	–	PR	–
Present report	1	Denosumab	-	PR	-

CR, complete response; PR, partial response; SD, stable disease; PD, progressive disease.

## 4 Conclusions

In this case report, we present the pulmonary metastases from a GCTB mimicking malignancy. PET/CT revealed a high accumulation of ^18^F-FDG in the lesions. A metastatic malignant tumor was suspected in this case; however, histological examination revealed a metastatic GCTB without malignant changes. Although the pulmonary nodules were unresectable, they were controlled with denosumab. Denosumab may be a treatment option for patients with unresectable GCTB metastases. Further studies on dose reduction or extension of the treatment interval of denosumab treatment are demanded.

## Data availability statement

The original contributions presented in the study are included in the article/supplementary material. Further inquiries can be directed to the corresponding author.

## Ethics statement

Ethical review and approval were not required for the study on human participants in accordance with the local legislation and institutional requirements. The patients/participants provided written informed consent to participate in this study. Written informed consent was obtained from the patients to publish any potentially identifiable images or data in this article.

## Author contributions

The manuscript was drafted by ShM, NY, and HT. ShM, NY, KH, AT, KI, YT, SeM and YA examined and treated the patient. TN performed the histopathological assessment. NY, TN, and HT supervised this study. All authors contributed to the article and approved the submitted version.
